# The APPLE Tree programme: Active Prevention in People at risk of dementia through Lifestyle, bEhaviour change and Technology to build REsiliEnce—randomised controlled trial

**DOI:** 10.1186/s13063-022-06557-6

**Published:** 2022-07-26

**Authors:** M. Poppe, L. Duffy, N. L. Marchant, J. A. Barber, R. Hunter, N. Bass, A. M. Minihane, K. Walters, P. Higgs, P. Rapaport, I. A. Lang, S. Morgan-Trimmer, J. Huntley, Z. Walker, H. Brodaty, H. C. Kales, K. Ritchie, A. Burton, J. Wenborn, A. Betz, C. Cooper

**Affiliations:** 1 UCL Division of Psychiatry, University College London, London, UK; 2 Department of Statistical Science, University College London, London, UK; 3 Research Department of Primary Care and Population Health, University College London, London, UK; 4 Norwich Medical School, University of East Anglia, Norwich, UK; 5 College of Medicine and Health, University of Exeter, Exeter, UK; 6 Centre for Healthy Brain Ageing, University of New South Wales, Sydney, Australia; 7 Department of Psychiatry and Behavioral Sciences, University of California, Davis, Sacramento, USA; 8 Institut de Neurosciences de Montpellier (INM), Montpellier, France; 9 Department of Behavioural Science and Health, University College London, London, UK; 10 Queen Mary University London, Centre for Psychiatry and Mental Health, Wolfson Institute for Population Health, London, UK

**Keywords:** Randomised controlled trial

## Abstract

**Background:**

Large-scale trials of multidomain interventions show that modifying lifestyle and psychological risk factors can slow cognitive decline. We aim to determine if a lower intensity, personally tailored secondary dementia prevention programme for older people with subjective or mild objective memory decline, informed by behaviour change theory, reduces cognitive decline over 2 years.

**Methods:**

A multi-site, single-blind randomised controlled trial recruiting 704 older adults at high dementia risk due to mild cognitive impairment (MCI) or subjective cognitive decline (SCD). Participants are randomised using 1:1 allocation ratio to the APPLE Tree intervention versus control arm (dementia prevention information), stratified by site. The intervention explores and implements strategies to promote healthy lifestyle, increase pleasurable activities and social connections and improve long-term condition self-management. Two facilitators trained and supervised by a clinical psychologist deliver ten, 1-h group video call sessions over 6 months (approximately every fortnight), video-call ‘tea breaks’ (less structured, facilitated social sessions) in intervening weeks and individual goal-setting phone calls every 2 weeks. From 6 to 12 months, participants meet monthly for ‘tea breaks’, with those not attending receiving monthly goal-setting phone calls. Participants receive a food delivery, pedometer and website access to cognitive training and information about lifestyle modification. Follow-ups for all outcome measures are at 12 and 24 months. The primary outcome is cognition (Neuropsychological Test Battery (NTB) score) at 24 months. Secondary outcomes are quality of life, cost per quality-adjusted life year (QALY) and wellbeing and lifestyle factors the intervention targets (diet, vascular risk, body weight, activity, sleep, anxiety, depression, social networks and loneliness, alcohol intake and smoking). Participants from purposively selected sites participate in qualitative process evaluation interviews, which will be analysed using thematic analytic methods.

**Discussion:**

If effective, the intervention design, involving remote delivery and non-clinical facilitators, would facilitate intervention roll-out to older people with memory concerns.

**Trial registration:**

ISRCTN17325135. Registration date 27 November 2019

**Supplementary Information:**

The online version contains supplementary material available at 10.1186/s13063-022-06557-6.

## Administrative information

Note: the numbers in curly brackets in this protocol refer to SPIRIT checklist item numbers. The order of the items has been modified to group similar items (see http://www.equator-network.org/reporting-guidelines/spirit-2013-statement-defining-standard-protocol-items-for-clinical-trials/).

**Table:** 

Title {1}	The APPLE Tree programme: Active Prevention in People at risk of dementia through Lifestyle, bEhaviour change and Technology to build REsiliEnce: Pilot of the intervention and Randomised Controlled Trial
Trial registration {2a and 2b}.	ISCRTN number: ISRCTN17325135; 10.1186/ISRCTN17325135
Protocol version {3}	Protocol version 5 (23/12/2020)
Funding {4}	This trial is funded by an Economic and Social Research Council/National Institute for Health Research programme grant (ES/S010408/1).
Author details {5a}	Queen Mary University London, Centre for Psychiatry and Mental Health, Wolfson Institute for Population Health: Claudia Cooper Claudia.cooper@qmul.ac.uk UCL Division of Psychiatry, University College London, London, UK: Michaela Poppe (m.poppe@ucl.ac.uk); Larisa Duffy (l.duffy@ucl.ac.uk); Natalie Marchant (n.marchant@ucl.ac.uk); Nick Bass (n.bass@ucl.ac.uk); Paul Higgs (p.higgs@ucl.ac.uk); Penny Rapaport (p.rapaport@ucl.ac.uk); Jonathan Huntley (j.huntley@ucl.ac.uk); Jennifer Wenborn (j.wenborn@ucl.ac.uk); Zuzana Walker (z.walker@ucl.ac.uk) Department of Statistical Science, University College London, London, UK: Julie Barber (j.barber@ucl.ac.uk) Research Department of Primary Care and Population Health, University College London, London, UK: Rachael Hunter (r.hunter@ucl.ac.uk); Kate Walters (k.walters@ucl.ac.uk) Department of Behavioural Science and Health, University College London, London, UK Alexandra Burton a.burton@ucl.ac.uk Norwich Medical School, University of East Anglia, Norwich, UK: Anne Marie Minihane (A.Minihane@uea.ac.uk); College of Medicine and Health, University of Exeter, Exeter, UK: Iain Lang(I.Lang@exeter.ac.uk); Sarah-Morgan-Trimmer (S.Morgan-Trimmer@exeter.ac.uk) Centre for Healthy Brain Ageing, University of New South Wales, Sydney, Australia: Henry Brodaty (h.brodaty@unsw.edu.au) Department of Psychiatry and Behavioral Sciences, University of California, Davis, Sacramento, USA: Helen Kales (hckales@ucdavis.edu) Institut de Neurosciences de Montpellier (INM), Montpellier, France: Karen Ritchie (karen.ritchie@inserm.fr)
Name and contact information for the trial sponsor {5b}	Joint Research Office, UCL Gower Street, London WC1E 6BT Email: uclh.randd@nhs.net
Role of sponsor {5c}	The study sponsor and funder have not been involved in study design; collection, management, analysis, and interpretation of data; writing of the report; or the decision to submit the report for publication. They will not have ultimate authority over any of these activities.

## Introduction

### Background and rationale {6a}

There is evidence from research studies that modifying lifestyle and psychological risk factors for dementia can delay and potentially prevent cognitive decline [1]. The Lancet Commission on dementia prevention, intervention and care identified potentially modifiable dementia risk factors that could be relevant in older people: cardio-metabolic dysfunction (diabetes and cardiovascular risks), physical inactivity, social isolation, hearing loss, depression, alcohol and smoking. They reported that 40% of dementia cases may be significantly associated with modifiable risk factors, which also included traumatic brain injury and air pollution [2]. A systematic review of predictors of cognitive decline and conversion to dementia among people with mild cognitive impairment (MCI) identified very similar risk factors [3].

In our systematic review of interventions for people aged 50+ with or without memory concerns, the most effective were group interventions, lasting at least 4 months, that promoted at least weekly activity and involved aerobic or resistance exercise and a cognitively stimulating or creative component; for some interventions, effects lasted up to a year beyond the intervention sessions [1]. Several major trials of multidomain interventions have produced contrasting results. The FINGER (Finnish Geriatric Intervention Study to Prevent Cognitive Impairment and Disability) trial demonstrated significantly improved cognition in participants aged between 60 and 77 years with vascular risk factors, who received a multi-component intervention (diet, exercise, cognitive training, vascular risk monitoring) relative to a control group over 2 years [4]. Results from other major trials are less promising. In the French Multidomain Alzheimer Preventive Trial (MAPT) [5], group sessions focused on diet, exercise and cognitive training (with addition of omega-3 supplementation) did not improve cognition in the main analysis, though a sub-analysis in people at high dementia risk (based on age and vascular risk) favoured the intervention. In the Dutch Prevention of Dementia by Intensive Vascular Care (PreDIVA) trial, in which vascular risk factors were targeted in people aged 70–78 without long-term conditions by a practice nurse, primary cognitive endpoints for effectiveness were not met [6].

These mixed results could be explained by the differences in sample populations. The FINGER trial population had on average greater cognitive impairment at baseline compared with the MAPT trial (MMSE score of 26 [4] versus 28 [5]), and in the preDIVA trial, a relatively healthy population was recruited. Alternatively, the contrasting trial results might be explained by differences in the interventions. Relative to MAPT and preDIVA, the FINGER intervention was more intensive and targeted a broader range of domains (see above). The Worldwide FINGERS collaboration is exploring how the FINGER intervention may be adapted to local contexts. This work will evaluate whether the effectiveness of the FINGER intervention is retained if it is adapted to reduce the amount of expert time (over 200 h per participant in the original intervention), which would be a barrier to widespread adoption, for example by using self-guided, internet-based delivery [4,7]. This work will find out whether briefer interventions can be as effective as the original FINGER intervention in populations at risk of dementia.

The initial stages of the APPLE tree programme have involved co-designing and piloting a secondary dementia prevention (APPLE Tree) intervention [8,9,10]. This is to our knowledge the first large trial of an intervention specifically aimed at people who are experiencing memory concerns. The group intervention involves setting goals to promote healthy diet and hydration, physical activity, engaging with life (increasing pleasurable activities), connecting with others (increase social connections), reducing alcohol and smoking, improving self-care of long-term physical conditions and improving sleep and mental wellbeing. There is a specific focus on managing anxieties associated with experiencing memory concerns and worries about dementia, which can feel like an illness in itself [8]. Here we report the protocol for a randomised trial to evaluate that intervention. Due to the Covid-19 pandemic, we adapted the originally planned face to face format to video-call groups and found the intervention was acceptable and feasible to conduct remotely in a pilot study [10].

We are recruiting older adults experiencing mild cognitive impairment (MCI) or subjective cognitive decline (SCD). MCI (objective cognitive symptoms and absence of dementia) affects 20% of people over 65. SCD (self-reported experience of memory problems without objectively impaired cognitive performance) affects a quarter to a half of people over 60 [11].

A core value of our APPLE Tree dementia prevention programme is inclusivity. We accounted for this in our recruitment strategy, which does not require potential participants to have contacted primary care about memory concerns. We are recruiting in areas selected for high ethnic density and socioeconomic deprivation. We are working with non-governmental organisations (NGOs) to recruit participants and to deliver the intervention and are mindful of the importance of recruiting a diverse group of facilitators, as we are delivering the intervention to a diverse group of participants. Some participating NGOs specifically work with older people from minority ethnic backgrounds. These strategies are designed to enrich the cohort in relation to socio-economically deprived and minority ethnic backgrounds, as these groups have higher rates of the principal dementia risk factors and dementia prevalence, but currently receive less preventative care [12].

### Objectives {7}

The primary objective is to evaluate the clinical effectiveness (in terms of reducing cognitive decline) of the APPLE Tree intervention in individuals with MCI or SCD over 24 months of follow-up. The secondary objectives are to calculate the cost per quality-adjusted life year (QALY) gained with the APPLE Tree intervention compared to the control, and to compare wellbeing and lifestyle factors targeted by the intervention (diet, vascular risk, body weight, activity, sleep, anxiety, depression, social networks and loneliness, alcohol and smoking) between groups over this period.

### Trial design {8}

This is a single-blind, multi-site, randomised controlled trial. Randomisation is blocked using a 1:1 allocation ratio to the APPLE Tree intervention versus control, stratified by site. Participants are followed up at 12 and 24 months; we will carry out a mixed methods process evaluation.

## Methods: participants, interventions and outcomes

### Study setting {9}

Participants are recruited through the English National Health Service (NHS). We are mailing study information to patients registered at participating primary care practices, who are aged 60 or over and have recorded risk factors for dementia (a frailty or diabetes code), inviting them to contact the study team if they are worried about their memory and interested in participating. Participating memory services will also invite potentially eligible participants to take part. Further recruitment is through the Join Dementia Research register of people interested in taking part in dementia studies, and NGOs for older people, including those collaborating in intervention delivery, who are advertising the study in online and face-to-face forums. We also advertise the study through the social media platform Twitter, the APPLE Tree study website and local and national newspapers. Outcome measures and the APPLE Tree intervention were intended to be conducted face-to-face. Due to Covid-19 restrictions, the study protocol was amended and assessments have been conducted via video call or telephone since participant recruitment commenced in October 2020. Where Covid restrictions permit, face-to-face outcome assessments are offered to participants who prefer this. Intervention groups are held remotely via video call. Study sites are listed here: https://www.ucl.ac.uk/psychiatry/research/mental-health-neuroscience-department/apple-tree/about-study.

### Eligibility criteria {10}

#### Inclusion criteria for participants with memory concerns

- Aged 60 years and above
- A score on the QuickMCI Screen [13] within educational and age normal ranges for MCI or SCD. O’Caomih et al. [13] reported that a cutpoint of <62 indicated objective cognitive impairment (MCI). We only include participants scoring 62+ who answered ‘yes’ to at least two of the following three questions, designed to detect SCD:

1. Has your memory deteriorated in the last 5 years? Or has a friend or family member noticed it deteriorating?
2. Is your memory persistently bad? Or has a friend or family member noticed it being persistently bad?
3. Are you concerned about this? Or are others around you concerned about this?

This approach is adapted from published measures of SCD [14,15]. Where researchers consider that QuickMCI scores may not be representative of a participant’s cognitive ability because English is their second language or they have few years of formal education, they discuss with the Chief Investigator (consultant old age psychiatrist), and exceptions are agreed on a case-by-case basis where context indicates results are consistent with MCI or SCD. Similarly, inclusion of any participant scoring <50 is discussed, as this may indicate undiagnosed dementia.

- Functional Assessment Questionnaire score of below 9 indicating no significant impairment related to the participant’s cognition [16].
- Having a relative, friend, or professional who is in at least monthly contact with the participant, and willing and has capacity to act as an informant

Individuals will be excluded if any of the following criteria apply:

- A diagnosis of dementia
- An AUDIT-C (Alcohol Use Disorders Identification Tool) score of 8+ which indicates hazardous or harmful use of alcohol [17]
- A diagnosed terminal condition
- Lacking capacity to consent at baseline
- Being in regular contact with a group facilitator (this could occur where NHS staff or NGOs are co-delivering the intervention, and risks contamination of the control arm)
- Insufficient understanding of spoken English, or a severe hearing impairment, such that they are unable to participate in the intervention (as judged by the participant).

### Who will take informed consent? {26a}

Trained researchers obtain written (or audio-recorded) informed consent from participants and informants at baseline. Capacity to consent at baseline is an inclusion criteria. Researchers ask who participants wish to be their personal consultee if they lose capacity during the trial. If researchers consider that a participant has lost capacity to decide whether to take part, they ask the participant’s preferred personal consultee whether they consider that the participant would have wished to continue to take part. If the personal consultee assents to the participant continuing in the study, they are asked to sign a consultee declaration form.

### Additional consent provisions for collection and use of participant data and biological specimens {26b}

Donation of blood (using a dried blood spot sample card) and saliva samples is an optional part of the trial. Intervention participants may opt to donate photographs shared in tea breaks to the study archive; if these include their image, they are asked to sign an additional consent form to indicate how they agree to it being used (for research, or for publication or display in an exhibition).

## Interventions

### Explanation for the choice of comparators {6b}

Participants in the control arm continue to receive usual care, which is determined by local treatment pathways. They are sent a brochure about dementia prevention published by the Alzheimer’s Society, which includes information about dementia risk factors, behavioural-change targets and signposting. This is a typical, usual intervention by memory services on diagnosing SCD or MCI in current NHS practice.

### Intervention description {11a}

The APPLE Tree manualised intervention has been co-designed with Public and Patient Involvement (PPI), academic, clinical, and older people’s advocacy group representatives. It is fully described elsewhere [10]. It comprises ten, 1-h group video-call sessions held fortnightly with 6–8 participants. A ‘tea break’ (i.e. unstructured, informal social sessions) is held for half an hour in the weeks in-between intervention sessions, with the same group. With allowance of breaks for public holidays, these twenty sessions span 5 to 6 months. Group sessions and goal calls focus on promoting: healthy diet and hydration, physical activity, engaging with life (increasing pleasurable activities), connecting with others (increase social connections), reducing alcohol and smoking and improving self-care of long-term physical conditions, sleep and mental wellbeing. Sessions are designed to be fun, informative and interactive. They include short video demonstrations of recipes in the intervention manual, and videos of activity suggestions. Participants are invited to bring healthy food and drinks to tea breaks and share photos illustrating their responses to the intervention and may choose to donate these to the study archive. Participants receive a phone call (up to 30 min) after each main session from a facilitator, who encourages them to set new goals or revise existing goals and record progress in their goal-setting booklet. Participants who have missed a session are offered a catch-up to discuss content of the session missed, usually combined with the goal phone call. We encourage participants to include relatives or friends to support them in their plans, and they can choose to invite them to the group sessions. Participants receive a single, non-perishable food delivery (oatmeal, tins of Cannellini, chickpeas and kidney beans, frozen berries, frozen salmon, brown pasta, extra virgin olive oil, passata, an aubergine, avocado, lemon juice, couscous, ground Cumin, fresh ginger, and if the participant has a freezer, frozen fish fillets of fish). These items are selected to support dietary advice (to follow a Mediterranean diet) and recipes suggested. Substitutes are provided where participants have food allergies, intolerances, preferences or no freezer.

Participants are sent the intervention manual and goal booklet prior to intervention and have access to the study website. This includes an online cognitive training battery, informed by current evidence [18]. It includes tasks to test working memory, prospective memory with pattern separation, processing speed, visuospatial attention and reasoning. Other resources to support wellbeing are available on the website. Any participants without a device are lent a Wi-Fi-enabled device to access the intervention via video call and the study website. Participants may include a pedometer in their plans to increase activity and set goals and are provided with one to use.

The intervention is delivered by two facilitators, and groups are organised so that participants share geographical proximity as far as possible. At non-NHS sites, it is delivered by a researcher from University College London (UCL) and a facilitator from an NGO working with older people. At NHS sites, it is delivered by two assistant psychologists. All facilitators are trained by role-play to deliver the intervention as defined in a facilitator manual by the research team (which includes health and clinical psychology, psychiatry, primary care and nutrition expertise). Facilitators attend group supervision with a clinical psychologist every fortnight, and there are additional individual supervision sessions as requested by facilitators or the study team. Monthly nutrition supervision is provided by a trained nutritionist. A researcher or third-sector worker provides additional technical support during groups, supporting participants with accessing video call and facilitators in showing videos.

### Criteria for discontinuing or modifying allocated interventions {11b}

So far, the intervention has been delivered remotely via video call due to Covid-19 restrictions. At the time of writing, there are no plans for face-to-face delivery of the intervention, as adherence is higher than expected. We are successfully facilitating novice users to participate in video calls, and they are willing to do so, though if this context changes, we may consider delivering some groups face to face. While we do not consider the intervention high risk, it is possible that intervention sessions may induce anxiety. If this occurs, facilitators can discuss this with participants during individual goal calls and explore whether they wish to continue with the groups. If a participant is consistently unable to adhere to the session ground rules (that ensure mutual respect and enjoyment of the groups), they may be asked to access the intervention through individual sessions only, and not to attend remaining group sessions.

### Strategies to improve adherence to interventions {11c}

Participants who miss intervention sessions are offered catch-up sessions. Participants are sent reminders about all intervention sessions.

### Relevant concomitant care permitted or prohibited during the trial {11d}

Trial participants in both arms continue to receive their usual health and social care. Information about prescribed medications, primary and community care services and emergency and hospital services accessed is recorded at baseline and follow-up data collection as part of the Client Service Receipt Inventory (CSRI).

### Provisions for post-trial care {30}

There are no provisions for post-trial care.

### Outcomes {12}

The *primary outcome* is cognition, measured using the Neuropsychological Test Battery (NTB) composite score (video call or face-to-face assessment) at 24 months. It is highly sensitive to change with excellent internal consistency and test-retest reliability [19]. It is validated for delivery by video call [20]. It comprises tests of memory, executive function, and paired associates learning and has nine validated components:

- Wechsler Memory Scale visual paired associates immediate recall (WMS-R) (score range, 0–18)
- Wechsler Memory Scale visual paired associates delayed recall (WMS-R) (range, 0–6)
- Wechsler Memory Scale logical memory immediate recall (WMS-IV) (score range 0–25)
- Wechsler Memory Scale logical memory delayed recall (WMS-IV) (score range 0–25)
- Wechsler Memory Digit Span—forwards (WAIS-IV) (score range, 0–16)
- Wechsler Memory Digit Span—backwards (WAIS-IV) (score range, 0–16)
- Rey Auditory Verbal Learning Test (RAVLT) immediate (RAVLT-I) (score range, 0–105)
- RAVLT delayed (RAVLT-D) (score range, 0–15)
- Controlled Word Association Test (COWAT)
- Category fluency test (CFT)

*Secondary outcome* measures are:

- Modified version of the Client Service Receipt Inventory (CSRI) for self-reported health and social care resource use [21]
- EuroQoL EQ-5D 5 Level (EQ-5D-5L) to measure preference-based health-related quality of life [22]
- Hospital Anxiety and Depression Scale (HADS) [23]
- Mediterranean Diet Adherence Score (MEDAS) assesses adherence the plant-based Mediterranean style diet intervention elements [24]
- MyFood24, a validated 24-h recall tool for dietary assessment [25], which in addition to capturing adherence to the APPLE Tree dietary goals, provides details of participants habitual diet. Participants provide details of all food and drink consumed the day before the assessment, and this is logged by researchers on the MyFood24 online tool.
- Pittsburgh Sleep Quality Index [26]
- Alcohol Use Disorders Identification Tool (AUDIT-C) to measure alcohol consumption [27]
- Primary support-network size [28]
- Brief loneliness scale [29]
- Smoking status (smoker versus non-smoker);
- Physiological measures including gait speed, grip strength [30], weight and body mass index (BMI),
- Physical activity and average heart rate (resting and high) over 2 weeks, using data downloaded from wearable sensors (Garmin) [31];
- Blood indices of dietary compliance (red blood cell omega-3 index and vitamin C); and cardiovascular and cognitive biomarkers of risk (plasma total, LDL and HDL cholesterol, triglycerides, glucose, HBA1c (as a measure of glucose tolerance), BDNF and insulin).

Informants will be asked to complete the following secondary measures:

- Functional Assessment Questionnaire measuring participants’ activities of daily living [16]
- General Health Questionnaire (GHQ-12) with regard to their psychological health [32].

All primary and secondary outcome measures are obtained at baseline, 12-month and 24-month follow-up. In addition, the MEDAS is collected at 6 months, to explore dietary changes in detail during and after the intervention.

#### Other covariates

We will collect a saliva sample at baseline for genome-wide genotyping using a global array to detect gene variants. We will use the Alzheimer’s disease polygenic risk score (including ApOE status) in the analysis. Participants will complete the List of Threatening Experiences [33], to measure concurrent life events, as these might have a significant effect on cognitive and mental wellbeing.

#### Process evaluation

This will comprise case studies at 2–4 intervention sites. At each site, we will conduct semi-structured, face-to-face, video call or telephone interviews with 8–10 participants, 3–5 relatives/ friends (study partners who have attended groups or otherwise supported participants) at 6 months, with a further brief interview at 12 months to investigate impacts during the less intensive stage of the intervention, between 6 and 12 months. All facilitators will be interviewed at 12 months, or sooner if they leave the project. Qualitative interviews will take place after quantitative outcomes have been collected at each time point to build on any reflections prompted by quantitative measures being administered. We will also conduct semi-structured, face-to-face or telephone interviews with 5–6 participants who leave the intervention prematurely.

The sampling strategy and topic guides are designed to test a priori theories about how the intervention works, and causal assumptions regarding intervention mechanisms that were developed during co-design and iterated by the team and PPI group (see Supplementary file for logic model). Interviews explore how participants experienced the intervention, how it might have supported them to make and maintain lifestyle changes, and their perceived impact of these, including on memory. We are, in addition to the main process evaluation, conducting 10–15 photo-elicitation interviews to explore the meanings participants attribute to photographs they donated to the study archive. Interviewees for these additional interviews are be purposively selected for ethnic diversity, as part of a sub-study exploring how dementia prevention messages are understood and experienced across different ethnic and cultural contexts.

We are also exploring how the intervention is implemented within the study (content/fidelity, frequency/duration, and coverage/reach). We are audio-recording one randomly selected group intervention session from each cohort to assess facilitator fidelity to the manual using a standard checklist which we have developed. We record attendance at intervention components, goals set and whether they are achieved using a standardised spreadsheet completed during goal phone calls, and information on use of cognitive training will be downloaded from the study website.

### Participant timeline {13}

Table 1 provides an overview of the timeline for enrolment, outcome assessments and intervention delivery. S_1-10_ refers to the 10 group intervention sessions, 6M, 12M and 24M refer to the follow-up assessments at 6, 12 and 24 months from baseline.

**Table 1:** Participant timeline

	Enrolment	Baseline													
**TIMEPOINT**	−*t*_1_	0	s_1_	s_2_	s_3_	s_4_	s_5_	s_6_	s_7_	s_8_	s_9_	s _10_	6M	12M	24M
**ENROLMENT:**															
Eligibility screening	X														
Informed consent		X													
Randomisation		X													
**INTERVENTION:**															
Sessions 1–10			X	X	X	X	X	X	X	X	X	X			
**ASSESSMENTS:**															
Demographics		X													
NTB		X												x	X
CSRI		X												X	X
EQ-5D-5L		X												X	X
FAQ		X												X	X
PSQI		X												X	X
HADS		X												x	X
MDS		X											X	X	X
24-h food recall		X												X	X
AUDIT		X												X	X
Smoking status		X												X	X
PSNS, LTE, BLS		X												X	X
GHQ-12		X												X	X
PM		X												X	X
PA		X												X	X
Bloods and saliva		X												X	X
Qualitative interviews (intervention arm)													X	X	

Legend: *AUDIT* Alcohol Use Disorders Identification Tool, *BLS* brief loneliness scale, *CSRI* Client Service Receipt Inventory, *EQ-5D-5L* EuroQoL EQ-5D 5 Level, *FAQ* Functional Assessment Questionnaire, *GHQ-12* General Health Questionnaire-12, *HADS* Hospital Anxie**t**y and Depression Scale, *LTE* list of threatening experiences, *MDS* Mediterranean Diet Score, *NTB* Neuropsychological Test Battery, *PSQI* Pittsburgh Sleep Quality Index, *PA* physical activity (step count) and resting heart rate (over 2 weeks), *PM* physiological measures (gait speed, grip strength, weight, body mass index, and hip and waist circumference), PSNS primary support network size

### Sample size {14}

The trial aims to recruit 704 participants (352 per randomised group). We calculated that this number is sufficient to detect, with 90% power and 5% significance, a difference of 0.15 in average NTB scores between intervention and control groups at 2 years (assumed standard deviation = 0.6) [34]. The calculation is based on an analysis that adjusts for baseline NTB score (assumed correlation coefficient between baseline and 24-month scores of 0.6) [34], intervention arm clustering (assuming an average of 36 participants per facilitator [32] after drop-out), an intracluster correlation coefficient of 0.03 and 10% drop-out. The initial calculation used unequal allocation (ratio 1:0.52), so that after inflation for clustering in the intervention arm, the allocation ratio is 1:1.

### Recruitment {15}

Recruitment sources are described in the study setting section {9}. Those interested in participating receive a Participant Information Sheet (PIS). A researcher then arranges an appointment to meet with them (video call or face-to-face) for a screening interview and, if they are eligible, elicit informed consent and conduct the baseline assessment.

We estimate that we will achieve recruitment of 704 participants within a 27-month period between October 2020 and December 2022. This equates to recruitment of 26 participants per month. In order to reach our recruitment target, participants are recruited through a range of recruitment sources, including primary care practices, memory services, NGOs for older people, the Join Dementia Research register, social media and the study website. We also advertise in local newspapers, and we are recruiting participants in response to an article about the study in a national newspaper.

## Assignment of interventions: allocation

### Sequence generation {16a}

Randomisation is at the level of the individual participant using a web-based application; Sealed Envelope, provided by Priment Clinical Trials Unit (CTU). Randomisation is blocked and stratified by site.

### Concealment mechanism {16b}

Details of treatment arm allocation are stored in a section of the Sealed Envelope database, to which blinded assessors do not have access. The trial manager and researchers involved in intervention procedures are informed of the randomisation outcome.

### Randomisation process {16c}

Researchers enrol participants in the Sealed Envelope database once the screening and baseline data has been collected. A member of the research team who is not blinded to treatment allocation then undertakes the randomisation. Participants are assigned to treatment groups through consecutive allocation of participant numbers and use of a Trial Participant Log.

## Assignment of interventions: blinding

### Who will be blinded {17a}

This is a single-blind study. Researchers conducting follow-up outcome measures are blind to participants’ treatment arm allocation. Study participants and their informants and researchers facilitating the intervention are aware of the participant’s treatment arm allocation due to the nature of the intervention. Statisticians are not be blinded, as due to clustering in the intervention arm, group allocations will be apparent when managing data.

### Procedure for unblinding if needed {17b}

There are no plans for intentional unblinding. In the unlikely event of a probable intervention-related SAE (serious adverse event), we will follow PRIMENT CTU procedures for unblinding. If a researcher becomes unblinded, the study team record this and ask an alternative blinded researcher to complete future outcome measures for that participant.

## Data collection and management

### Plans for assessment and collection of outcomes {18a}

Researchers assess outcome measures at baseline, 12 months and 24 months post-randomisation. In addition, the MEDAS is conducted for all participants at 6 months, as part of a sub-study to explore dietary changes in greater depth. A full list of primary and secondary outcome measures is provided under ‘Outcomes {12}’, and timelines are outlined in Table 1. Outcome measures and interviews are conducted by researchers fully trained in the study measures and conducting qualitative interviews.

### Plans to promote participant retention and complete follow-up {18b}

Each participant is offered a £20 gift voucher after completion of each outcome assessment (baseline, 12- and 24-month follow-ups) to thank them for their time. A further £20 gift voucher is offered to each participant taking part in a process evaluation qualitative interview. If required, the outcome measures are conducted over two separate visits. Participants who indicate that they do not have time to complete follow-up have the option to complete the primary outcome only.

### Data management {19}

Data is collected from participants manually on trial-specific paper Case Report Forms (CRFs) that are stored securely at UCL and entered into the database, managed by Sealed Envelope. Sealed Envelope has been assessed by the Priment CTU to ensure that adequate processes are in place and are being followed for quality management, software and security. Intervention sessions and qualitative interviews are recorded using a password-protected, encrypted recorder, and files are transferred between professional transcription services and UCL via a secure server hosted by the transcription service. All identifiable information is removed from the transcripts including any reference to names or places. Audio files are password-protected and stored in separate folders to the transcripts on secure servers and only accessible to those authorised to use the files.

### Confidentiality {27}

The CRFs do not bear the participant’s name. Personal data needed to re-contact participants is held on secure servers. Consent forms are held manually in locked filing cabinets on University premises, and only named research team members have access.

### Plans for collection, laboratory evaluation and storage of biological specimens for genetic or molecular analysis in this trial/future use {33}

Samples are collected by participants at home, and we provide instructions and appropriate equipment. Dried bloodspot sample cards are posted to UCL and stored at −80 °C and saliva samples at room temperature.

## Statistical methods

### Statistical methods for primary and secondary outcomes {20a}

Statistical analyses will be described in a predefined detailed statistical analysis plan agreed prior to commencement of data analysis and conducted according to ICH E9 and PRIMENT CTU standard operating procedures [35].

## Summary of baseline data and flow of trial participants

Participants’ baseline characteristics will be described by treatment group using summary statistics (means (standard deviations), medians (with interquartile ranges), counts and proportions, as appropriate). A CONSORT diagram will describe the flow of participants through the trial including numbers eligible, randomised, and consenting and with data available for the primary outcome (http://www.consort-statement.org/). The number of participants withdrawing from the intervention and the study and reasons where supplied will be reported.

## Primary outcome analysis

For each randomised group, we will summarise the primary outcome (NTB scores at 24 months) using means with standard deviations and graphically examine the distribution of the score. The effect of the intervention will be described using the between-group difference in means calculated with a 95% confidence interval. This estimate will be obtained from a mixed-effects multiple regression model adjusting for baseline NTB score and site and allowing for facilitator clustering in the intervention arm [36]. The intracluster correlation (with 95% confidence interval) will be calculated to describe the extent of facilitator clustering. All analyses will be carried out on an intention-to-treat basis comparing the groups as randomised using all available data.

NTB scores at 12 months will also be summarised by treatment group using descriptive statistics. Profile plots will be used to illustrate trends in the average scores over time (baseline, 12 months and 24 months). The mixed-effects model will be extended to include the 12-month measurement and to consider the effect of the intervention over time by including a treatment-group-by-time interaction.

## Secondary outcome analysis

Secondary outcomes will be analysed using similar models to those described for the primary outcome.

## Fidelity analysis

We will audio-record one randomly selected group intervention session from each cohort to assess facilitator fidelity to the manual using a standard checklist which we have developed. We will report session attendance. We will report adherence as the proportion of participants who have attended (or had a catch-up for) at least five main sessions (moderate attendance) as well as those attending or catching-up all ten main sessions. We will also report the number of main sessions (or catch-ups) attended, using appropriate summary statistics, and the relationship between number of sessions attended and treatment effect (measured on the primary outcome) in the intervention arm.

## Economic evaluation

The economic analysis will evaluate the cost-effectiveness of the APPLE Tree intervention compared to the control over 24 months from an English NHS and Personal Social Services (PSS) perspective, using patient-level trial data. The principal analysis will be the incremental cost per quality-adjusted life year (QALY) gained with the APPLE Tree intervention compared to the control from a health and social care cost perspective. A secondary analysis will report the incremental cost per unit change in NTB.

Health and social care resource use will be collected via participant-completed questionnaires administered at baseline, 12 months and 24 months asking about the previous 12 months for care home accommodation and the previous 6 months for other health and social care resource use, using a modified version of the CSRI questionnaire [21]. Resource use will be costed using nationally published sources, including the Personal Social Services Research Unit (PSSRU) [37], NHS reference costs [38] and the British National Formulary (BNF) [39]. The cost of the intervention, applied to the intervention group only, will include the cost of training the facilitators, supervision, organising and delivering the sessions and any items purchased for the purposes of the intervention. QALYs will be calculated based on participant responses to the EQ-5D-5L captured via participant-completed questionnaires at baseline, 12 months and 24 months, using the area under the curve method and utility scores obtained from relevant UK tariffs. Costs and utilities from 12 to 24 months will be discounted at a rate of 3.5%. The mean difference in costs and QALYs between the two groups will be calculated using regression analysis adjusting for baseline, site and including clustering for facilitator in the intervention arm, with 95% confidence intervals calculated using bootstrapping.

To align the economic evaluation with the primary outcome, the primary analysis for the economic evaluation will be complete case with sensitivity analyses adjusting for predictors of missingness and multiple imputation (see section ‘Methods in analysis to handle protocol non-adherence and any statistical methods to handle missing data {20c}’). Two-part bootstrapping adjusting for baseline, site and including clustering will be used to construct cost-effectiveness acceptability curves of the probability that the intervention is cost-effective compared to the control for a range of cost-effectiveness thresholds for a QALY gained and unit change in NTB. Additional sensitivity analyses will be performed, where relevant, to assess the impact of alternative modelling assumptions on the results, including projecting 6-month resource use collected at 12 and 24 months over the full 12-month time-period between follow-ups.

## Process evaluation

We will use reflexive thematic analysis to analyse qualitative interviews [40], and NVivo 12 to manage the data. The qualitative analysis will be underpinned by a critical realist perspective, using a deductive lens based on theory-development work (see logic model in Supplementary file) and research questions about how the intervention operates to produce its outcomes, but also taking an inductive approach to identify novel findings. Researchers will systematically code transcripts into meaningful fragments and label these initial codes, discussing and resolving discrepancies [40]. Two researchers will independently code 10% of transcripts and compare coding to ensure consistency of approach. We will then develop themes responding to research questions. We will triangulate qualitative findings with fidelity ratings and 12-month quantitative outcomes, using a joint display table [41], to evaluate putative mechanisms of intervention effect. Process evaluation findings will refine our conceptual understanding of how the intervention works, building on theory developed in the APPLE Tree programme [8,9,10].

### Interim analyses {21b}

There are no plans to conduct interim analyses, as the intervention is considered low risk.

### Methods for additional analyses (e.g. subgroup analyses) {20b}

The following supportive analyses will be carried out for the primary and secondary outcomes using the same modelling approaches as described previously:

- Estimation of an unadjusted treatment effect.
- Estimation of the treatment effect adjusting for any concerning imbalances in baseline characteristics.
- Exploratory analysis (as sample size is calculated as sufficient for main analyses only) of whether MCI diagnosis versus SCD diagnosis (defined by Quick MCI cutpoint at baseline) moderates intervention effectiveness.

### Methods in analysis to handle protocol non-adherence and any statistical methods to handle missing data {20c}

Where there is missing outcome data, the main analyses will be based on available data, relying on an assumption that data is missing at random. Reasons for missingness will be described, and frequency (%) of subjects with missing data, by reason, will be provided for each randomised group (and for each outcome). Characteristics of participants with and without missing outcome data will be compared using logistic regression models (with missing yes/no as the outcome) to identify characteristics associated with missingness. In a sensitivity analysis, the treatment effect will be re-estimated with additional adjustment for baseline predictors of missingness. Further sensitivity analyses based on multiple imputation methods will be considered if appropriate.

### Plans to give access to the full protocol, participant-level data and statistical code {31c}

The protocol can be accessed via ISRCTN registry (ISRCTN17325135; 10.1186/ISRCTN17325135). Participant-level data and statistical code are available from the corresponding author upon reasonable request.

## Oversight and monitoring

### Composition of the coordinating centre and trial steering committee {5d}

The study is overseen by a Project Management Group (PMG) and an independent Trial Steering Committee (TSC). The PMG includes the Chief Investigator and trial manager, all co-applicants and two PPI group representatives. The group meets twice a year and sends updates to co-investigators. The PMG review recruitment figures, SAEs (serious adverse events) and substantial amendments to the protocol prior to submission to the REC. All site Principal Investigators (PIs) are kept informed of substantial amendments.

The TSC consists of an independent chair, independent statistician, two PPI representatives, Priment CTU representative, the CI, trial manager and lead statistician. The TSC will recommend any appropriate amendments/actions for the trial as necessary.

### Composition of the data monitoring committee, its role and reporting structure {21a}

The Data Monitoring Committee is combined with the TSC because the intervention is considered low risk.

### Adverse event reporting and harms {22}

Any SAEs that are classed as related and unexpected will be reported to the ethics committee and Priment CTU, according to Priment CTU standard operating procedures.

### Frequency and plans for auditing trial conduct {23}

We conduct site initiation visits prior to research sites being opened and monitor sites during the trial. We record protocol deviations and report these to the sponsor and TSC. Annual progress reports are sent to the REC.

### Plans for communicating important protocol amendments to relevant parties (e.g. trial participants, ethical committees) {25}

Where there are significant changes to our trial documentation, including the protocol, we seek an ethics amendment. The trial manager sends amendments to the sponsor for approval before submitting it to the ethics committee, notifies all researchers and research sites of the amendment submissions and outcomes and provides amended documents to sites.

### Dissemination plans {31a}

We will disseminate our findings in peer-reviewed journals and at an international conference and on the ISCTRN registry. We will present findings in local forums for health and social care professionals. Participants who have indicated on the consent form they are interested in the results will be sent a lay summary of the findings on project completion. Participants donating photographs to the archive have the option to give permission for their display in exhibitions. We plan to develop this archive into a coproduced photography exhibition to explore the lived experiences of memory loss and dementia prevention.

## Discussion

The APPLE Tree intervention is one of several large trials testing potentially scalable multidomain approaches to dementia prevention that, if successful, could make a real-world difference to dementia prevalence in whole populations. We had developed a relatively short (15 h + follow-ups) face to face group intervention prior to the pandemic, and piloted it in a form adapted for group video calls [10]. Previous remotely delivered dementia prevention interventions have been individually delivered, using internet platforms or telephone contact, and such interventions are yet to demonstrate effectiveness [42]. The HATICE trial, yet to report, is testing an interactive Internet platform, designed to encourage lifestyle changes with the remote support of a lifestyle coach to improve cardiovascular health, with cognition as a secondary outcome [43].

To our knowledge, APPLE Tree is the first group-based multidomain dementia prevention remote intervention to be trialled. In the pilot study, the group component, with peer learning and social connectedness, was valued [10]. There is a risk that an internet-delivered intervention excludes participants who are digitally excluded or not comfortable with technology, who may be at greatest need. Of the 4 million UK adults who had never used the internet in 2019, over half were aged 75 years and over [44]. We developed strategies to engage participants through working collaboratively with study partners, providing initial individual help to get online, and lending device to those without computers. These have successfully engaged many novice internet users; for example, only three of our ten pilot participants had used video calling before the intervention [10].

We are working with the NHS and NGO partners to produce an effective national implementation approach, so that if our multidomain lifestyle and behaviour intervention improves cognition, it is useful and used. We plan a pre-implementation study to explore how the intervention may be implemented successfully beyond the trial sites if it is effective.

### Trial status

The current version of the protocol is version number 5 dated 23/12/2020. The first participant was recruited on 21/10/2020. Participant recruitment is ongoing (we randomised 300 participants by November 2021) and the project recruitment is projected to end December 2022.

## Supplementary Information

**Additional file 1.**

## Abbreviations

APPLE: Tree Active Prevention in People at risk of dementia through Lifestyle, bEhaviour change and Technology to build REsiliEnce
AUDIT-C: Alcohol Use Disorders Identification Tool
BDNF: Brain-derived neurotrophic factor
BMI: Body mass index
BNF: British National Formulary
CFT: Category fluency test
CI: Chief Investigator
COWAT: Controlled Word Association Test
CRFs: Case Report Forms
CSRI: Client Service Receipt Inventory
CTU: Clinical trials unit
EQ-5D-5L: EuroQol
GDPR: General Data Protection Regulation
GHQ-12: General Health Questionnaire
GP: General Practitioner
FINGER: Finnish Geriatric Intervention Study to Prevent Cognitive Impairment and Disability
HADS: Hospital Anxiety and Depression Scale
HBA1c: Haemoglobin A1C
HDL: High density lipoprotein
ICC: Intracluster correlation coefficient
ICH: International Conference on Harmonization
ISRCTN: International Standard Randomised Controlled Trial Number
LDL: Low-density lipoproteins
MCI: Mild cognitive impairment
MDS: Mediterranean Diet Score
NHS: National Health Service
NTB: Neuropsychological Test Battery
PI: Principal Investigator
PIS: Participant Information Sheet
PMG: Project Management Group
PPI: Public and Patient Involvement
PSQI: Pittsburgh Sleep Quality Index
PSS: Personal Social Services
PSSRU: Personal Social Services Research Unit
QALY: Quality-adjusted life year
RAVLT: Rey Auditory Verbal Learning Test
REC: Research Ethics Committee
SAE: Serious adverse event
SCD: Subjective cognitive decline
TSC: Trial Steering Committee
UCL: University College London
UEA: University of East Anglia
WMS: Wechsler Memory Scale

## References

[1] Whitty E, Mansour H, Aguirre E, Palomo M, Charlesworth G, Ramjee S, Poppe M, Brodaty H, Kales HC, Morgan-Trimmer S, Nyman SR, Lang I, Walters K, Petersen I, Wenborn J, Minihane AM, Ritchie K, Huntley J, Walker Z, Cooper C (2020). Efficacy of lifestyle and psychosocial interventions in reducing cognitive decline in older people: systematic review. Ageing Res Rev.

[2] Livingston G, Huntley J, Sommerlad A, Ames D, Ballard C, Banerjee S (2020). Dementia prevention, intervention, and care: 2020 report of the Lancet Commission. Lancet..

[3] Cooper C, Sommerlad A, Lyketsos CG, Livingston G (2015). Modifiable predictors of dementia in mild cognitive impairment: a systematic review and meta-analysis. Am J Psychiatry.

[4] Rosenberg A, Mangialasche F, Ngandu T, Solomon A, Kivipelto M (2020). Multidomain interventions to prevent cognitive impairment, Alzheimer's disease, and dementia: from FINGER to World-Wide FINGERS. J Prev Alzheimers Dis.

[5] Andrieu S, Guyonnet S, Coley N, Cantet C, Bonnefoy M, Bordes S, Bories L, Cufi MN, Dantoine T, Dartigues JF, Desclaux F, Gabelle A, Gasnier Y, Pesce A, Sudres K, Touchon J, Robert P, Rouaud O, Legrand P, Payoux P, Caubere JP, Weiner M, Carrié I, Ousset PJ, Vellas B (2017). MAPT Study Group. Effect of long-term omega 3 polyunsaturated fatty acid supplementation with or without multidomain intervention on cognitive function in elderly adults with memory complaints (MAPT): a randomised, placebo-controlled trial. Lancet Neurol.

[6] Hoevenaar-Blom MP, Richard E, Moll van Charante EP (2021). Association of targeting vascular risk factors with a reduction in dementia incidence in old age: secondary analysis of the Prevention of Dementia by Intensive Vascular care (preDIVA) randomized clinical trial. JAMA Neurol.

[7] Heffernan M, Andrews G, Fiatarone Singh MA, Valenzuela M, Anstey KJ, Maeder A (2019). Maintain your brain: protocol of a 3-year randomized controlled trial of a personalized multi-modal digital health intervention to prevent cognitive decline among community dwelling 55 to 77 year olds. J Alzheimers Dis.

[8] Cooper C, Aguirre E, Barber JA, Bass N, Brodaty H, Burton A, Higgs P, Hunter R, Huntley J, Lang I, Kales HC, Marchant NL, Minihane AM, Ritchie K, Morgan-Trimmer S, Walker Z, Walters K, Wenborn J, Rapaport P (2020). APPLE-Tree (Active Prevention in People at risk of dementia: Lifestyle, bEhaviour change and Technology to REducE cognitive and functional decline) programme: Protocol. Int J Geriatr Psychiatry.

[9] Poppe M, Mansour H, Rapaport P, Palomo M, Burton A, Morgan-Trimmer S, Carter C, Roche M, Higgs P, Walker Z, Aguirre E, Bass N, Huntley J, Wenborn J, Cooper C (2020). “Falling through the cracks”; stakeholders' views around the concept and diagnosis of mild cognitive impairment and their understanding of dementia prevention. Int J Geriatr Psychiatry.

[10] Cooper C, Mansour H, Carter C, Rapaport P, Morgan-Trimmer S, Marchant NL, et al. Social connectedness and dementia prevention: pilot of the APPLE-Tree video-call intervention during the Covid-19 pandemic. Dementia (London). 2021:14713012211014382. 10.1177/14713012211014382 Epub ahead of print. PMID: 33913362. 10.1177/14713012211014382 PMC8679164 33913362

[11] Röhr S, Pabst A, Riedel-Heller SG (2020). Estimating prevalence of subjective cognitive decline in and across international cohort studies of aging: a COSMIC study. Alz Res Therapy.

[12] Cooper C, Lodwick R, Walters K (2015). Observational cohort study: Deprivation and access to anti-dementia drugs in the UK. Age Ageing.

[13] O'Caoimh R, Timmons S, Molloy DW (2016). Screening for mild cognitive impairment: comparison of “MCI specific” screening instruments. J Alzheimers Dis.

[14] Jessen F, Amariglio RE, Buckley RF, van der Flier WM, Han Y, Molinuevo JL (2020). The characterisation of subjective cognitive decline. The Lancet Neurology.

[15] Jessen F, Amariglio RE, van Boxtel M, Breteler M, Ceccaldi M, Chetelat G (2014). A conceptual framework for research on subjective cognitive decline in preclinical Alzheimer's disease. Alzheimer's & dementia: the journal of the Alzheimer's Association.

[16] Pfeffer RI, Kurosaki TT, Harrah CH, Chance JM, Filos S (1982). Measurement of functional activities in older adults in the community. J Gerontol.

[17] Khadjesari Z, White IR, McCambridge J, Marston L, Wallace P, Godfrey C, Murray E (2017). Validation of the AUDIT-C in adults seeking help with their drinking online. Addict Sci Clin Pract.

[18] Corbett A, Owen A, Hampshire A, Grahn J, Stenton R, Dajani S, Burns A, Howard R, Williams N, Williams G, Ballard C (2015). The effect of an online cognitive training package in healthy older adults: an online randomized controlled trial. J Am Med Dir Assoc.

[19] Harrison J, Minassian SL, Jenkins L, Black RS, Koller M, Grundman M (2007). A neuropsychological test battery for use in Alzheimer disease clinical trials. ArchNeurol..

[20] Munro Cullum C, Hynan LS, Grosch M, Parikh M, Weiner MF (2014). Teleneuropsychology: evidence for video teleconference-based neuropsychological assessment. Journal of the International Neuropsychological Society : JINS.

[21] Beecham J, Knapp M, Thornicrost C, Brewin C, Wing J (2001). Costing psychiatric intervention. Measuring mental health needs.

[22] Wolfs CA, Dirksen CD, Kessels A, Willems DC, Verhey FR, Severens JL (2007). Performance of the EQ-5D and the EQ-5D+C in elderly patients with cognitive impairments. Health Qual Life Outcomes.

[23] Bjelland I, Dahl AA, Haug TT, Neckelmann D (2002). The validity of the Hospital Anxiety and Depression Scale: an updated literature review. J Psychosom Res.

[24] Martinez-Gonzalez MA, Garcia-Arellano A, Toledo E, et al. A 14-item Mediterranean diet assessment tool and obesity indexes among high risk subjects: the PREDIMED trial. PLoS One. 2012;7(8). 10.1371/journal.pone.0043134 PMC3419206 22905215

[25] Wark PA, Hardie LJ, Frost GS (2018). Validity of an online 24-h recall tool (myfood24) for dietary assessment in population studies: comparison with biomarkers and standard interviews. BMC Med.

[26] Buysse DJ, Reynolds CF, Monk TH, Berman SR, Kupfer DJ (1989). The Pittsburgh Sleep Quality Index: a new instrument for psychiatric practice and research. Psychiatry Res.

[27] Saunders JB, Aasland OG, Babor TF, Delafuente JR, Grant M (1993). Development of the Alcohol-Use Disorders Identification Test (Audit) - WHO Collaborative Project on Early Detection of Persons with Harmful Alcohol-Consumption .2. Addiction..

[28] Brugha TS, Morgan Z, Bebbington P (2003). Social support networks and type of neurotic symptom among adults in British households. Psychol Med.

[29] Hughes ME, Waite LJ, Hawkley LC, Cacioppo JT (2004). A short scale for measuring loneliness in large surveys: results from two population-based studies. Research on Aging.

[30] Op Het Veld LPM, de Vet HCW, van Rossum E, Kempen GIJM, van Kuijk SMJ, Beurskens AJHM (2018). Substitution of Fried's performance-based physical frailty criteria with self-report questions. Arch Gerontol Geriatr.

[31] Massoomi MR, Handberg EM (2019). Increasing and evolving role of smart devices in modern medicine. Eur Cardiol.

[32] Goldberg DP, Williams P (1988). A user’s guide to the General Health Questionnaire.

[33] Brugha T, Bebbington P, Tennant C, Hurry J (1985). The list of threatening experiences - a subset of 12 life evnt categories with considerable long-term contextual threat. Psychol Med.

[34] Ngandu T, Lehtisalo J, Solomon A, Levalahti E, Ahtiluoto S, Antikainen R, et al. A 2 year multidomain intervention of diet, exercise, cognitive training, and vascular risk monitoring versus control to prevent cognitive decline in at-risk elderly people (FINGER): a randomised controlled trial. Lancet. 2015. 10.1016/S0140-6736(15)60461-5 25771249

[35] Topic ICH. E 9 Statistical Principles for Clinical Trials. EMEA Sept. 1998; (https://www.ema.europa.eu/en/documents/scientific-guideline/ich-e-9-statistical-principles-clinical-trials-step-5_en.pdf).

[36] Walwyn R, Roberts C (2010). Therapist variation within randomised trials of psychotherapy: implications for precision, internal and external validity. Stat Methods Med Res.

[37] Curtis L, Burns A. Unit Costs of Health and Social Care 2020. Personal Social Services Research Unit, University of Kent, Canterbury. 2020. 10.22024/UniKent/01.02.84818.

[38] England NHS (2020). National Schedule of Costs. National Cost Collection for the NHS.

[39] Joint Formulary Committee: British National Formulary (online) London: BMJ Group and Pharmaceutical Press. http:// www.medicinescomplete.com (2020).

[40] Braun V, Clarke V (2019). Reflecting on reflexive thematic analysis. Qualitative Research in Sport, Exercise and Health.

[41] Bazeley P (2018). Integrating analyses in mixed methods research.

[42] Whitfield T, McConnell B, Renouf P, Mansour H, Zabihi S, Aguirre E, Walker Z, Cooper C, Marchant NL (2021). The effect of remotely delivered lifestyle interventions on cognition in older adults without dementia: a systematic review and meta-analysis. Ageing Res Rev.

[43] Richard E, Jongstra S, Soininen H (2016). Healthy ageing through internet counselling in the elderly: the HATICE randomised controlled trial for the prevention of cardiovascular disease and cognitive impairment. BMJ Open.

[44] Munanga A (2019). Cybercrime: a new and growing problem for older adults. J Gerontol Nurs.

