# Supplementary material for: The APPLE Tree programme: Active Prevention in People at risk of dementia through Lifestyle, bEhaviour change and Technology to build REsiliEnce—randomised controlled trial
Source: Trials. 2022 Jul 26;23:596. doi: 10.1186/s13063-022-06557-6 (PMC9315085; doi:10.1186/s13063-022-06557-6)
Supplement: Supplementary file 1 — Additional file 1. [file 13063_2022_6557_MOESM1_ESM.docx]

**Outputs**

10 main sessions 10 tea breaks (+ 6 monthly light-touch engagement tea-breaks)

10 goal phone calls

Use of cognitive training weekly

Goals set and met

Participants connect e.g. Whatsapp group, contact information, in-person meet-up

APPLE-Tree Book of wisdom

**Activities**

**Diet:**

**Capability**: information, recipe suggestions from facilitators and peers

**Opportunity**: food delivery, planning how to change diet (eg help from family)

**Motivation**: videos, peers bring food to tea break, peers share pictures in the tea break

**Physical health:**

**Capability**: information, debunking health myths

**Opportunity**: activity planning

**Motivation**: peer feedback

**Engagement:**

**Capability**: attendance

**Opportunity**: facilitated discussions, activity diary and reflection, problem solve activity schedule

**Motivation**: conversation with peers, role modelling

**Mental wellbeing:**

**Capability**: information, relaxation activity

**Opportunity**: relaxation exercise script and CD, activity diary

**Motivation**: practice in session, peer recommendation and experience/feedback

**Exercise:**

**Capability**: information from peers and facilitators

**Opportunity**: planning (activity diary), pedometer

**Motivation**: videos, peer recommendation and experience/feedback

**Mechanisms**

352 intervention participants receive:

food delivery, pedometers Manuals

Goal booklets

Website with cognitive training/Eden app/ other resources

Facilitators support participants to access zoom – including mifi enabled tablet is needed

Non-Clinical facilitators from UCL and third-sector sites receive group training course (12 days) from team

**PRIMARY OUTCOME: Improved cognitive function at 2 years**

**Figures 2-4: Logic models link activity/ goal attainment to outcomes**

Decreased loneliness & increased social network size

Improved sleep and decreased anxiety/depression symptoms

Improved self management of long term conditions

Decreased risk of mental illness, CVD, Type II diabetes, stroke

**Outcomes**

**Resources**

Contexts: pandemic, post-pandemic, online delivery, third sector facilitators liminal state prior to intervention, food culture, cultural expectations/practices of gender

**Figure 1: Overview logic model**

**Figure 2: Logic model linking engagement/ mental wellbeing goals to outcomes**

**Engagement components, activities:**

S1: Ground rules

S3: Introduction to activity diary

S4: Review activity diary

S5: Review activity diary

S8: Finding activities

S10: Future planning

Cognitive training use

**Mental Wellbeing components, activities:**

S1: Maximising your health (motivation)

S3: Using values to aid motivation

S4: Review activity diary

S5: Sleep and review activity diary

S8: Breathing Space exercise, anxiety and avoidance S9: Anxiety and memory

decreased loneliness, increased social network size

Decreased risk of Alzheimer’s disease/ improved cognition & functioning

**Outcomes> Goal Attainment**

**Mechanisms > Goal Attainment**

Increased social engagement pleasant activities/events

Engagement with preventative/ primary care

Increased use of relaxation techniques

Increased mental/ physical/ social activity/ participation

Improved sleep

Improved wellbeing (lower anxiety or better mood)

Decreased risk of falls/ fractures

decreased depression/ anxiety symptoms

**Intervention goals**

**Figure 3: Logic model linking physical exercise/ health goals to outcomes**

Eden app

Increased aerobic exercise (increased step count)

Decreased risk of CVD, Type II diabetes, lower HbA1c, insulin, cholesterol, LDL

Decreased risk of Alzheimer’s disease/ improved cognition & functioning

**Outcomes> Goal Attainment**

**Mechanisms > Goal Attainment**

Better self-management of Long Term Conditions

Engagement with preventative/ primary care

Increased mental/ physical/ social activity/ participation

Increased strength-based / flexibility exercise

**Moderate physical activity target** to attain 100 minutes of weekly moderate

intensity activity by 2 months (bronze), 120 minutes, by 4 months (silver), and 150 minutes after 6 months (gold)

Improved sleep

Improved wellbeing (lower anxiety or better mood)

Decreased risk of falls/ fractures

decreased depression/ anxiety symptoms

Decreased risk of stroke

**Intervention goals**

**Figure 4: Logic model linking nutrition goals to outcomes**

**Intervention goals**

**Outcomes> Goal Attainment**

**Mechanisms > Goal Attainment**

Vitamin D supplementation

Increased B12/B6/ folate

Increased fish intake, Omega-3 supplementation

Increased wholegrains & fibre

Decreased salt intake

Decrease total carbohydrate intake, Decreased sugar intake

prevent dehydration

Decreased risk of CVD, Type II diabetes, lower HbA1c, insulin

Increase polyphenol consumption (including flavonoids) (antioxidant/ anti-inflammatory compounds) and vitamin C

Decreased LDL cholesterol

Increased fruit, vegetables, legumes, herbs, seeds, spices (switch to red wine)

Increase nuts, olive oil

Decreased saturated fats (less dairy, red meat)

to reduce to <14 alcohol units per week over 3+ days, smoking cessation

Target increases on MDS

Improved sleep

Decreased risk of stroke

Decreased risk of falls/ fractures

decreased depression & anxiety symptoms

Increased Omega-3

Prevent Vitamin D deficiency

Decreased risk of Alzheimer’s disease/ improved cognition

Lower GI index

Weight loss in those with BMI above ideal range
